# Laser Irradiation-Induced DNA Methylation Changes Are Heritable and Accompanied with Transpositional Activation of *mPing* in Rice

**DOI:** 10.3389/fpls.2017.00363

**Published:** 2017-03-21

**Authors:** Siyuan Li, Qiong Xia, Fang Wang, Xiaoming Yu, Jian Ma, Hongping Kou, Xiuyun Lin, Xiang Gao, Bao Liu

**Affiliations:** ^1^Key Laboratory of Molecular Epigenetics of MOE, Northeast Normal UniversityChangchun, China; ^2^School of Life Sciences, Jilin Agricultural UniversityChangchun, China; ^3^College of Oceanology & Food Science, Quanzhou Normal UniversityQuanzhou, China; ^4^College of Agronomy, Jilin Agricultural UniversityChangchun, China; ^5^Jilin Academy of Agricultural SciencesChangchun, China

**Keywords:** Nd^3+^YAG laser irradiation, DNA methylation, transposable elements, epigenetic inheritance, rice

## Abstract

DNA methylation is an integral component of the epigenetic code in most higher eukaryotes. Exploring the extent to which DNA methylation can be altered under a specific condition and its heritability is important for elucidating the biological functions of this epigenetic modification. Here, we conducted MSAP analysis of rice plants with altered phenotypes subsequent to a low-dose Nd^3+^YAG laser irradiation. We found that all four methylation patterns at the 5′-CCGG sites that are analyzable by MSAP showed substantial changes in the immediately treated M0 plants. Interestingly, the frequencies of hypo- and hypermethylation were of similar extents, which largely offset each other and render the total methylation levels unchanged. Further analysis revealed that the altered methylation patterns were meiotically heritable to at least the M2 generation but accompanied with further changes in each generation. The methylation changes and their heritability of the metastable epigenetic state were verified by bisulfite sequencing of portion of the retrotranspon, *Tos17*, an established locus for assessing DNA methylation liability in rice. Real-time PCR assay indicated that the expression of various methylation-related chromatin genes was perturbed, and a Pearson correlation analysis showed that many of these genes, especially two AGOs (*AGO4-1* and *AGO4-2*), were significantly correlated with the methylation pattern alterations. In addition, excisions of a MITE transposon, *mPing*, occurred rampantly in the laser irradiated plants and their progenies. Together, our results indicate that heritable DNA methylation changes can be readily induced by low-dose laser irradiation, and which can be accompanied by transpostional activation of transposable elements.

## Introduction

DNA methylation is an important epigenetic marker that occurs frequently at cytosine bases, and plays important roles in orchestrating gene expression and maintaining genome stability. In plants, cytosine methylation occurs in three different sequence contexts: CG, CHG, and CHH (H is any nucleotide except G), but CG methylation stands as the most predominant pattern (Lister et al., 2008). A large body of studies have indicated that although DNA cytosine methylation was relatively stable and transgenerationally inheritable, it can be perturbed to change under certain conditions (Kou et al., 2011; Ou et al., 2012).

DNA methylation level and pattern for a given organism are the results of dynamic interplay between methylation and demethylation. An established function of cytosine methylation at promoter regions is to repress gene expression transcriptionally, and methylation within the gene body is involved in alternative splicing (Wang et al., 2016). In plants, *de novo* methylation is controlled by an RNA-dependent methylation (RdDM) pathway (Matzke and Mosher, 2014). Once established, DNA methylation is maintained through various mechanisms depending on sequence context. DNA methytransferase 1 (MET1, homolog of animal Dnmt1) and chromomethylases (CMT3, a plant-specific DNA methytransferase) target and maintain CG and CHG methylations, respectively (Law et al., 2010). Null mutation of the MET1-coding gene cause genome-wide loss of ^m^CGs and pleiotropic developmental defects both in *Arabidopsis* and rice (Stroud et al., 2013; Hu et al., 2014). RdDM mainly targets euchromatic regions and is excluded from pericentrometric heterochromation regions surrounding centromeres (Zemach et al., 2013). DNA methylation in heterochromatic sequences preferentially require a plant-specific SWI2/SNF2-like chromatin-remodeling protein called DDM1 (Decrease in DNA Methylation 1). The mutation of DDM1 leads to strong transcriptional activation of transposable elements (TEs) (Lippman et al., 2004).

Active DNA demethylation plays important roles in the gene regulation, and the 5-methycytosine (5-meC) can be demethylated passively during cell replication, or actively due to action of bi-functional DNA glycosylases that not only recognize and remove 5-meC from dsDNA, but also show lyase activity (Law et al., 2010). In rice, active DNA demethylation is initiated by the REPRESSOR OF SILENCING 1 (ROS1), DEMETER (DEM), and DEMETER-LIKE (DML) family (Ooi and Bestor, 2008).

In plants, the total 5-^m^C contents vary extensively among species, ranging from 6% in *Arabidopsis* to 30% in tobacco, which is probably related to differences in genome size (C-value) and abundance of TEs and other types of repetitive sequences (Alonso et al., 2015). TEs account for 35% of the rice genome, and many of which are retrotransposons, including roughly 14% LTR and 1% non-LTR retrotransposons, respectively. Some TEs in the rice genome, including both DNA transponsons and retrotransposons, are still capable of reactivation at the transcriptional or transpositional levels. TE activation at both levels may have phenotypic consequences by influencing expression of their neighbor genes or insertional mutagenesis (Lisch, 2013). Ample evidence indicates that DNA methylation is a major mechanism repressing TE activity under normal conditions, while loss of methylation due to genetic or environmental perturbations may lead to their de-repression (Wessler, 1996; Kashkush and Khasdan, 2007; Lisch, 2013).

The miniature inverted-repeat transposable elements (MITEs) are a kind of TEs, which predominate numerically in the rice genome (Feschotte et al., 2002; Casacuberta and Santiago, 2003; Jiang et al., 2004). The miniature *Ping* (*mPing*) (Feschotte et al., 2002; Kikuchi et al., 2003; Nakazaki et al., 2003; Jiang et al., 2004) is a quiescent MITE transopson in most rice cultivars under natural growing conditions, but was found to be activated by strong perturbing conditions like tissue culture and hydrostatic pressurization (Lin et al., 2006; Ngezahayo et al., 2009). Being devoid of the transposase-encoding sequences, the mobility of *mPing* is commonly dependent on other autonomous transposons, such as *Ping* and *Pong*, to provide the transposase required for its transposition (Feschotte et al., 2002; Kikuchi et al., 2003; Nakazaki et al., 2003; Jiang et al., 2004).

*Tos17*, a Ty1-Copia class I autonomous LTR retrotransposon, is one of the few active retrotransposons identified in the rice genome (Hirochika et al., 1996). *Tos17* has two copies in the rice reference genome (cv. Nipponbare) (Sabot, 2014), and both copies are silenced under normal conditions. However, the *Tos17* copy located on chromosome 7 can be activated to transpose by tissue culture and other conditions (Ding et al., 2007; Ou et al., 2015). Rampant mobilization of *Tos17* was detected in rice lines derived from introgressive hybridization with *Zizania latifolia* (Wang H. Y. et al., 2010). The body region of *Tos17* is generally accepted as an established locus in the rice genome for assaying cytosine methylation liability (La et al., 2011).

Heavy-ion beam irradiation and Ethyl methanesulfonate (EMS) induced mutagenesis were frequently used for breeding proposes and molecular biology (Serrat et al., 2014; Hirano et al., 2015). Low-dose laser irradiation has been successfully used in boosting crop performance, including promoting germination, growth and yield, positively influence physiological and biochemical parameters, and improving the disease resistance (Xu et al., 2008; Liu et al., 2016). Being an unusual condition, we suspect that low-dose laser irradiation might also influence epigenetic stability of the treated plants. Indeed, Wang H. et al. (2010) found that laser irradiation induced cytosine methylation changes in sorghum, and the alteration frequency in the inter-line F1 hybrids was higher than that of their pure-line parents, suggesting an interaction of hybridity and laser irradiation. Nevertheless, no information is yet available regarding if cytosine DNA methylation patterns and transposon activity could be affected subsequent to laser irradiation, and for this purpose, rice is the testing plant of choice. The present study was designed to address this issue.

Here, we report that low-dose laser irradiation induced extensive changes in DNA methylation patterns in rice plants that showed phenotypic alterations. The methylation changes correlate with altered expression of some of the chromatin-related genes according correlation analysis. Importantly, *mPing* was mobilized in the methylation-altered plants and their progenies. Transgenerational analysis indicated that the altered methylation patterns could be inherited to organismal progenies. Together, our results indicate that low-dose laser irradiation could readily induce heritable epigenetic changes and transpostional activity of TEs in plants.

## Materials and methods

### Plant materials and laser irradiation

A pure-line rice cultivar, Jinongda18 (Ma et al., 2003) was used in this study. Uniform germinating seeds were pretreated with 355, 532, and 1,064 nm Nd^3+^ YAG pulsed laser for 6, 10, 15, 20 times, respectively (Table 1). Eleven treatment groups with different cumulative doses of laser irradiation were set, and the untreated wild-type (WT) plants were used as the mock control. More than 1000 plants were used for the mock and 11 stress treatments, and the treated seeds were allowed to continue germinating in petri-dishes for 5d in a growth cabinet (30°C during the day and 25°C during the night, 16/8 h photoperiod at 50 μmolm^−2^s^−1^). Seedlings were then transferred to a homogeneous experimental paddy-field plot at Jilin Agricultural University, Changchun, China, in accord with season. Plant showing prominent phenotypic changes especially in plant height and heading date were tagged. The heading date was recorded as the first plant showing panicle emergence. Five plants were randomly chosen to measure their height. A single treated M0 individual with the shortest height and the latest heading date, designated as M0#11, was selected to generate the M1 progeny by self-pollination, then, a single M1 individual, designated as M1#1, was chosen to produce the M2 generation by self-pollination.

**Table 1:** **Phenotypes for plant height and heading date of WT (untreated) and various groups of laser-treated rice**.

**Groups**	**Device**	**Power (kv)**	**Wavelength (nm)**	**Exposure frequency (min)**	**Plant height (cm)**	**Heading date (days)**
WT^#^	–	–	–	–	101 ± 1.58	84
1	Nd^3+^YAG	4	355	10	102 ± 2.12	83
2	pulsed			15	100 ± 3.08	84
3	laser			20	98 ± 3.39	84
4			532	6	101 ± 2.45	83
5				10	102 ± 2.00	84
6				15	99 ± 2.24	87
7				20	95 ± 2.12^**^	88
8			1,064	6	100 ± 2.24	87
9				10	99 ± 2.24	87
10				15	95 ± 1.58^**^	88
11^##^				20	93 ± 3.00^**^	89

Data were presented as t mean ± standard deviation, ^*^P < 0.05,

** *P < 0.01*.

# *WT of rice cultivar Jinongda18*.

## *M0-11 was chosen for transgenerational study*.

### Methylation-sensitive amplified polymorphism (MSAP) analysis

The flag-leaves of rice plants in WT group and the 11 low-dose laser-treated groups were sampled as pools, while the M0#11, M1, and M2 progeny were sampled as individuals for DNA isolation at the grain filling stage. DNA was isolated using the modified CTAB method and purified by phenol extractions. MSAP is a modified version of the amplified fragment length polymorphism (AFLP) to detect the stability and alteration in cytosine DNA methylation at the 5′-CCGG sites (Yaish et al., 2014). Genomic DNA was digested with *Eco*RI combined with *Hpa*II or *M*s*p*I (New England Biolabs, Beverly, Massachusetts), ligated with *Eco*RI and H/M adapters, and then amplified with one pair of pre-selective and 20 pairs of selective primers (Supplementary Table 1). The amplification products of MSAP were resolved by 5% denaturing polyacrylamide gel electrophoresis and visualized by silver staining. Only clear and completely reproducible bands in two independent experiments were scored.

### Rational for MSAP data tabulation

MSAP is performed using *Hpa*II and *Msp*I, a pair of isoschizomers that recognize the same restriction site (5′-CCGG) but have different sensitivities to methylation of the cytosines. *Hpa*II will not cut if either of the cytosines is fully (double-strand) methylated, whereas *MspI* will not cut if the external cytosine is fully- or hemi- (single-strand) methylated (McClelland et al., 1994). Thus, for a given DNA sample, H0M1 indicates CG methylation; H1M0 indicates CHG methylation; H1M1 indicates no methylation; and H0M0 indicates CG/CHG methylation. Based on the principles, the changes of MSAP patterns were divided into four categories: (a) CG hyper: H1M1 to H0M1, H1M0 to H0M0; (b) CHG hyper: H1M1 to H1M0, H0M1 to H0M0; (c) CG hypo: H0M1 to H1M1, H0M0 to H1M0; and (d) CHG hypo: H1M0 to H1M1, H0M0 to H0M1.

### Isolation and sequencing of variant MSAP bands

Bands showing alteration in methylation patterns in the MSAP profiles in the laser-irradiated M0 plants and/or their progenies relative to WT were isolated, boiled with ddH_2_O, and amplified with the pre-selective primers used in the original MASP analysis. The PCR products were purified with Wizard PCR Preps Purification System (Promega). Then the purified PCR products were ligated into pM18-T vector (Promega), and then sequenced. Homology analysis was performed by BlastX at the NCBI website (http://www.ncbi.nlm.nih.gov/). Sequence alignment was done by the CLUSTALW program using the Genedoc software.

### Real-time reverse transcriptase (RT)-PCR analysis

Total RNA was isolated from the flag-leaves at the same developmental stage as that used for DNA methylation analysis by the Trizol Reagent (Invitrogen). Then RNA was treated with DNaseI (Invitrogen), reverse-transcribed by the SuperScript TMRNase H-Reverse Transcriptase (Invitrogen), and subjected to qRT-PCR analysis using gene-specific primers (Roche LightCycler 480). Genes encoding DNA methytransferase1 or *MET1* (LOC_Os07g08500, DMT707), *CMT3* (LOC_Os05g13790, DMT703), *DRM2-1* (LOC_Os03g02010, DMT706), and *DRM2-2* (LOC_Os05g04330, DMT710), *DME1* (LOC_Os01g011900, DNG702), *DME2* (LOC_Os05g37350, DNG701), *DDM1* (LOC_Os03g51230, CHR741), Argonaute *AGO1-1* (LOC_Os02g45070, AG0711), *AGO1-2* (LOC_Os04g47870, AG0708), *AGO4-1* (LOC_Os04g06770, AG0705), *AGO4-2* (LOC_Os01g16870, AG0703) were analyzed in WT, M0, M1, and M2 progeny to interrogate the impacts of laser irradiation on their staedy-state transcript abundance. These gene-specific primers were designed by the Primer Premier 5 software (Supplementary Table 2). A β-actin gene of rice (LOC_Os05g0438800) was used as an internal control. DNA contamination was tested by inclusion of RNAs without RT. Three batches of independently isolated RNAs were used as biological replications. The relative amounts of the gene transcripts were determined using the Ct (threshold cycle) method and the fold-change data were analyzed by the 2^−ΔΔCt^ method.

### Locus-specific PCR assay on *mPing* excision

Based on the principle that presence vs. absence of *mPing* produces 433 bp difference in length in given locus, excision of mPing from a known locus can be easily detected by locus-specific PCR amplification. Thus, a set of 53 pairs of locus-specific primers was designed by the Primer Premier 5 software (Supplementary Table 3), and each pair of primers bracketed an intact *mPing* based on the rice reference genome sequence of the standard laboratory genotype Nipponbare (http://rgp.dna.affrc.go.jp). The amplicons were visualized by ethidium bromide staining after electrophoresis through 2% agarose gels. A set of empty donor sites for *mPing* excision were identified, isolated and sequenced, together with their corresponding *mPing*-containing loci.

### Transposon display

Transposon display (TD) was performed by combining the *MseI*-adaptor-specific primers either with a *mPing* subterminal-specific primer or with a set of inter-simple sequence repeat (ISSR) exactly as previously reported (Jiang et al., 2003). Major procedures, i.e., digestion, ligation, pre-selective amplication, selective amplication were the same as for MSAP. And the restriction enzyme *MseI* (New England Biolabs) was used, the pre-selective primers were *MseI* +0 (5′- GATGAGTCCTGAGTAA) and *mPing* internal amplification primer (5′- GCTGACGAGTTTCACCAGGATG), the selective primers were 8 *MseI* +2 (Supplementary Table 4) and *mPing* proximal ends amplification primers (5′- TGTGCATGACACACCAGTG). The novel bands in TD gels of laser irradiated plants (M0, M1, and M2) relative to WT were considered as putative *mPing de novo* insertions.

### Southern blot analysis

Genomic DNA (3 μg per lane) of the various plants was digested by *Hin*dIII or *Xba*I (New England Biolabs). Digested DNA was run through 1% agarose gel and transferred onto Hybond-N^+^ nylon membranes (Amersham Pharmacia Biotech, Piscataway, New Jersey) by the alkaline transfer recommended by the supplier. And the *mPing* (positions: 6–430), *Pong* (BK000586.1), and *Ping* (AB087616.1) probes were prepared as previously described (Lin et al., 2006). The probe-fragments were gel-purified and labeled with fluorescein-11-dUTP by the Gene Images random prime-labeling module (Amersham Pharmacia Biotech). Hybridization signal was detected by the Gene Images CDP-Star detection module (Amersham Pharmacia Biotech). The filters were exposed to X-ray films.

### Bisulfite sequencing of a mark locus

Genomic DNA was modified using an EZ DNA Methylation-Gold kit (Zymo Research) according to the manufacturer's recommendations. Modified DNA was purified using a Zymo-Spin IC column (Zymo Research). The primers of retrotransposon *Tos17*, an established locus in the rice genome for assessing DNA methylation liability, for bisulfite sequencing were designed using the MethPrimer program (http://www.urogene.org/methprimer/) (Supplementary Table 5). For each PCR, 1.0–3.0 μl of bisulfite-treated DNA was used, and the PCR products were cloned into the pMD18-T vector and sequenced. More than 20 clones were sequenced for each sample. Analyses of the bisulfate sequencing results were conducted at the Kismeth website (http://katahdin.mssm.edu/kismeth). The methylation levels per site (CG, CHG, and asymmetric CHH) were calculated by dividing the number of non-converted (methylated) cytosines by the total number of cytosines within the assay (Ngezahayo et al., 2009).

### Statistics

Statistical significance was determined using SPSS 11.5 for Windows (http://www.spss.com/statistics/). Continuous variables e.g., plants height, fold-change data in qRT-PCR were presented as the mean ± standard deviation and statistically tested by an unpaired, two-tailed *t*-test. A value of *P* < 0.05 was considered significant and *P* < 0.01 was very significant. The Pearson correlation analysis between the DNA methylation pattern variations (detected by MASP) and the expression levels of methylation-related genes (detected by qRT-PCR) was calculated by the range method using SPSS. −1 ≦ Pearson's *r* ≦ 1. Pearson's *r* > 0 indicates the two variables have a positive correlation, Pearson's *r* < 0 indicates the two variables have a negative correlation, Pearson's *r* = 0 indicates the two variables have no correlation. The color depth symbolizes the values of Pearson's r, and the red and blue blocks denote positive and negative correlations, respectively.

## Results

### Plant growth inhibition by the laser irradiation treatments

**Figure 1:**
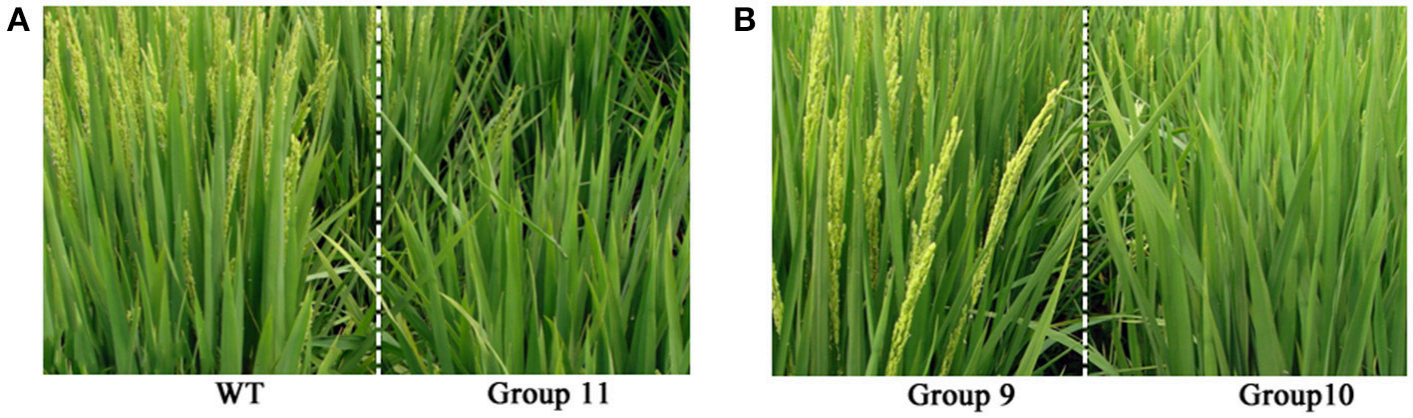
**Phenotypic changes in the M0 generation of Jinong 18 after low-dose laser-irradiation. (A)** WT vs. Group 11; **(B)** Group 9 vs. Group 10.

Phenotyping was conducted at the reproduction stage after the laser treatment in rice cultivar Jinongda 18. Phenotypic variations especially plant height and heading dates were observed in all the 11 laser treatment groups with variable extents (Table 1). Of 1,000 laser irradiated germinating seeds, *ca*. 990 plants grew to maturity in the experimental paddy-field. The heading date was recorded as the first plant showing panicle emergence in one group, and it was 84 days in WT, and which was delayed in most laser-treated groups. For example, heading date was 88 days in groups 7 and 10, and 89 days in group 11 (Figure 1). These observations indicated that long-wave laser treatment was more effective than short-wave laser, and the treatment frequency was a key factor which influences the degree of phenotypic variations in the same wavelength.

Five plants were randomly chosen to quantitatively measure the plant height, and the mean plant height was 101 ± 1.58 cm in WT (Table 1), and the common feature was dwarfing in all the 11 laser-treated groups. For example, it was 95 ± 2.12 cm in group 7 (*P* < 0.01), 95 ± 1.58 cm in group 10 (*P* < 0.01), the shortest height was 93 ± 3.00 in group 11 (*P* < 0.01). It indicated that laser irradiation negatively influenced plant growth and development, and the degrees of impact were largely dose-dependent.

### Alteration of cytosine methylation patterns in the laser-treated rice plants and its transgenerational inheritance

**Figure 2:**
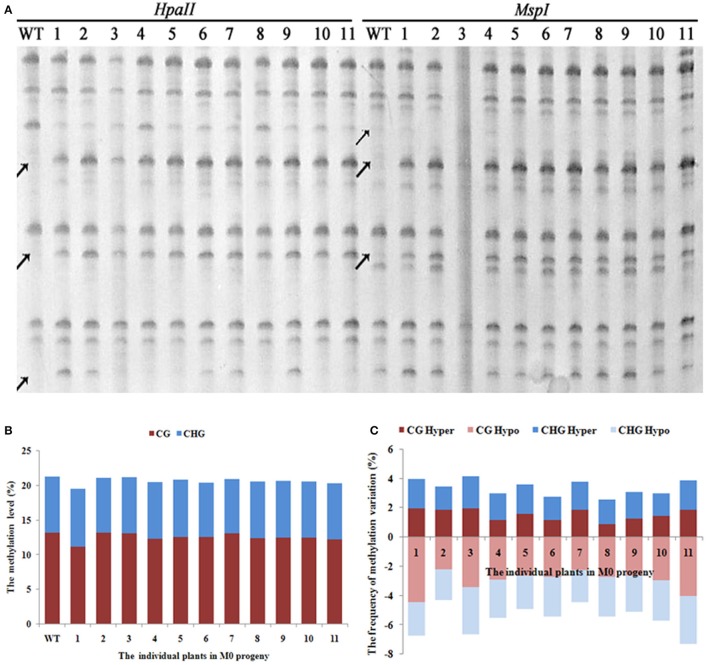
**(A)** Examples of MSAP gels and **(B)** the total methylation levels **(C)**, variations occurred in CG and CHG sites in M0 generation after laser-irradiation. The arrows shows methylation variations.

A total of 1,140 clear and reproducible bands (between two technical replicates, starting from the first step, i.e., DNA isolation) were scored using 20 pairs of selective primers in the MSAP analysis (Supplementary Table 1) for the 11 selected M0 individuals that showed clear phenotypic variations, along with WT (Figure 2A). The tabulated methylation level was 21.2% (^m^CG + ^m^CHG) in WT, and all but one (M0-1) of the 11 M0 plants showed similar levels of methylation to that of WT, which collectively ranged from 19.4 to 21.1% (Figure 2B). This suggests that the laser irradiation treatments did not cause large changes in total DNA methylation level at the 5′-CCGG sites. Notably, however, for the single M0 plant (M0-1) that did show clear loss of methylation, it mainly occurred at the CG sites (Figure 2B). In contrast to the general stability in methylation level, we found that all four methylation patterns (CG hyper, CG hypo, CHG hyper, and CHG hypo) that are discernible based on the MSAP data (Materials and methods) showed significant changes in all 11 laser irradiated M0 plants relative to WT. The frequencies of these methylation pattern alterations ranged from 1 to > 4% (Figure 2C). The similar frequencies of hyper vs. hypo methylation changes at both CG and CHG sites clearly have offset each other and render the total methylation levels broadly constant (Figure 2B). Notably, in all the MSAP profiles we did not find parallel band changes between the two isoschizomers, *Hpa*II and *Msp*I, indicating nucleotide sequence changes at the 5′-CCGG sites should be rare if occurred at all in these low-dose laser treated rice plants (Figure 2A).

**Figure 3:**
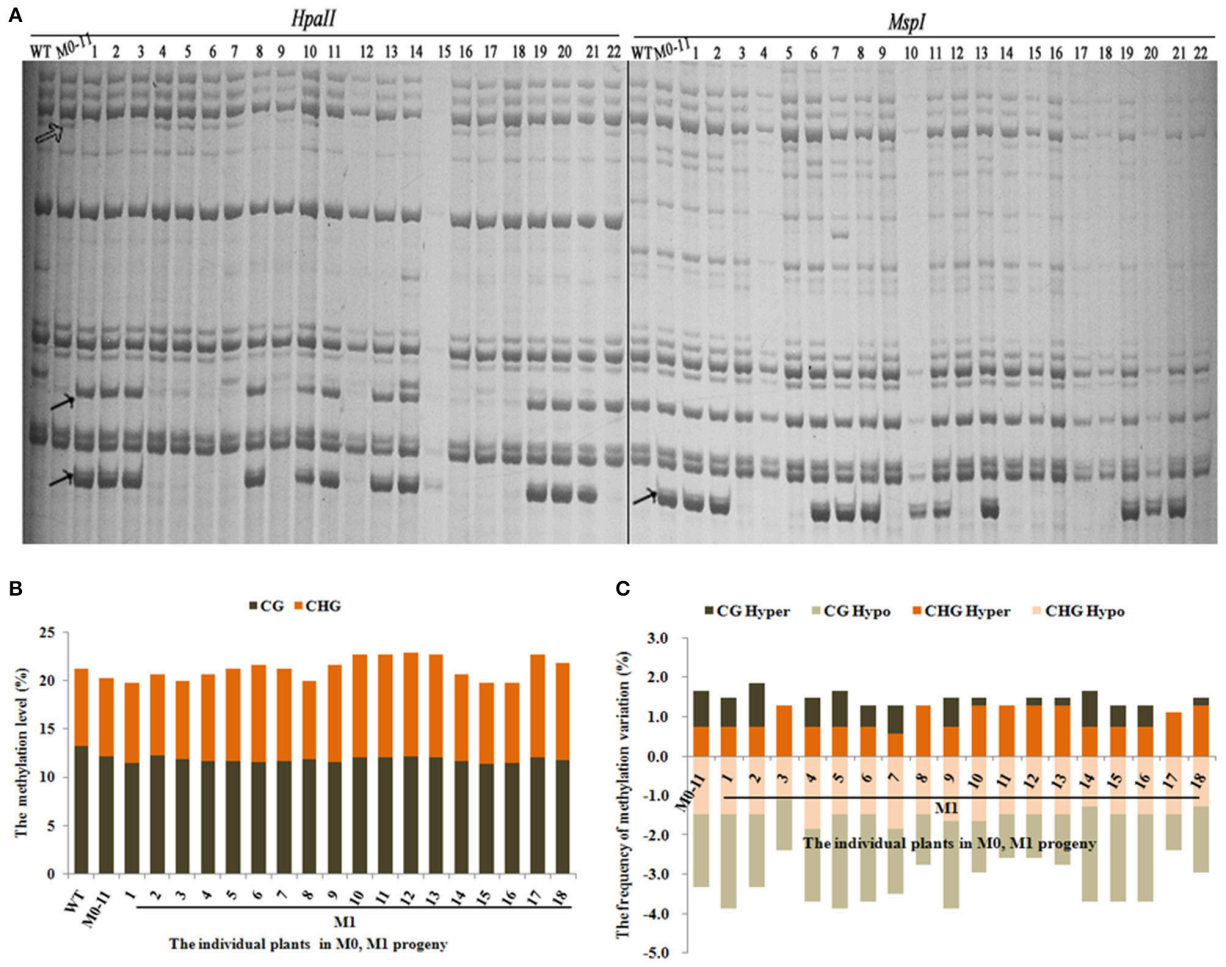
**(A)** Examples of MSAP gels and **(B)** the total methylation levels, **(C)** variations occurred in CG and CHG sites in M1 generation after laser-irradiation. The arrows shows methylation variations.

Next, we asked whether the methylation changes were inheritable to the next organismal generation. For this purpose, we chose plant M0-11 which showed the most obvious phenotypic changes (Table 1) to produce the M1 generation via self-pollination. A total of 543 reproducible bands (between two technical replicates) were scored using 10 informative selective MSAP primers in 18 randomly chosen M1 plants (Figure 3A). Results showed that the total methylation level of M0#11 was 20.3%, and which ranged from 19.7 to 22.8% in the M1 plants (Figure 3B), suggesting a moderate fluctuation in methylation level occurred in the M1 plants, which contrasted with the broad methylation level stability among the M0 plants (Figure 2B). Analysis of the four methylation patterns revealed the important observation that a great majority of the altered methylation patterns in the M0 plant (M0-11) was inherited to all 18 plants of the M1 generation (Figure 3A). Nevertheless, it is important to note that heritability of the alterations was clearly much less than 100% and variable across the 18 M1 individuals (Figure 3C). Moreover, gaining of additional methylation pattern alterations, especially CG hypo and both CG hyper and CHG hyper patterns, were apparent in most of the M1 plants (Figure 3C). The less than 100% heritability, together with unbalanced gain vs. loss of additional alteration, is consistent with the discernible fluctuations in total methylation level in the M1 plants (Figure 3B).

**Figure 4:**
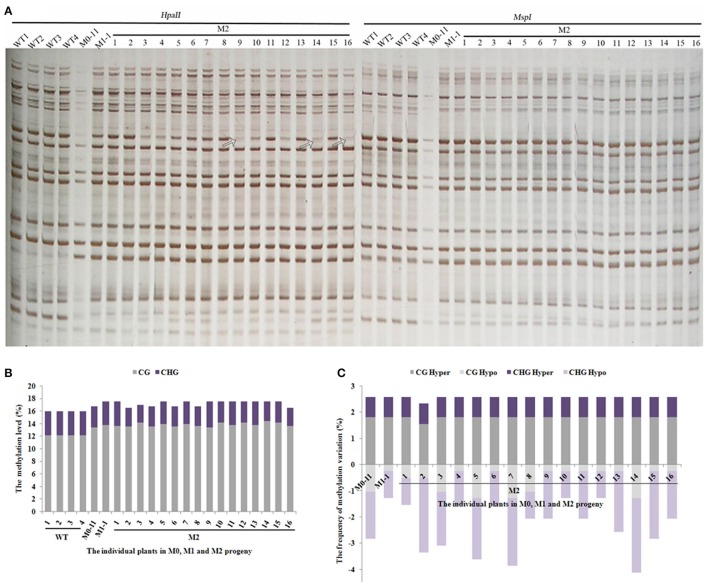
**(A)** Examples of MSAP gels and **(B)** the total methylation levels, **(C)** variations occurred in CG and CHG sites in M2 generation after laser-irradiation. The arrows shows methylation variations.

The still dynamic nature of methylation patterns in the M1 plants promoted us to address the issue further. We thus chose a single M1 plant (M1-1) to produce the M2-generation plants by self-pollination. A total of 543 reproducible bands (between two technical replicates) were scored by using seven pairs of selective MSAP primers across 16 randomly selected M2 individuals (Figure 4A). Results indicated that the methylation levels of all 16 M2 plants remained similar to that of the M1 plant (Figure 4B). Importantly, except for one M2 plant (M2-2), the two hyper-methylation patterns (CG hyper and CHG hyper) were uniform and the same as those of M0-11 and M1-1 (Figure 4C), pointing to their stabilization. By contrast, the two hypomethylation patterns (CG hypo and CHG hypo) were still as fluctuating among the M2 plants (Figure 4C) as they were among the M1 plants (Figure 3C). Together, our results suggest that although some of the newly acquired methylation pattern alterations due to the treatment were inherited and stabilized by the M2 generation, DNA methylation patterns in general are still dynamic in the M2 generation.

### Validation of DNA methylation alteration and its transgenerational dynamics by locus-specific bisulfite sequencing

**Figure 5:**
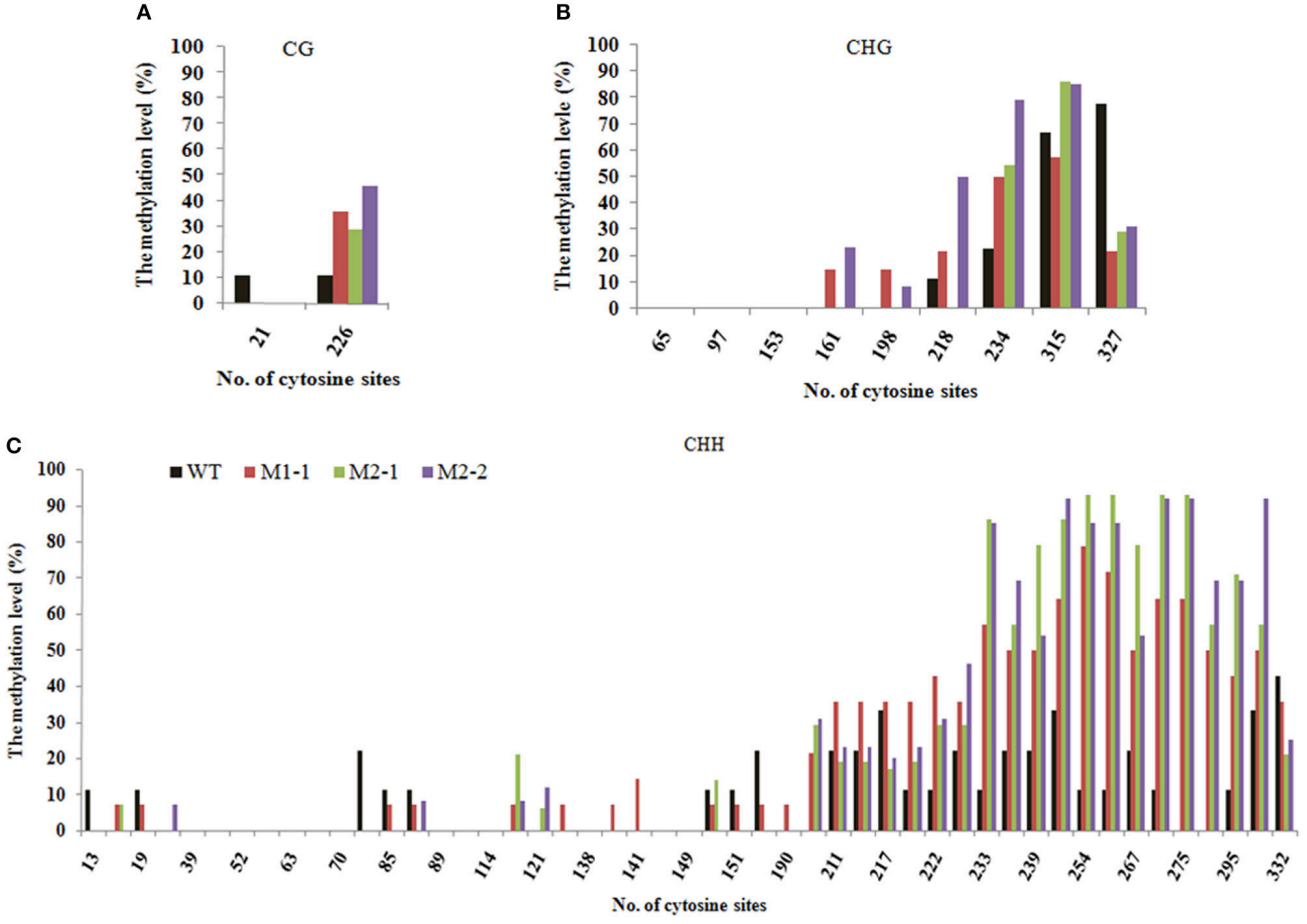
**The Bisulfite sequencing of portion of the retrotransposon *Tos17* (5′-LTR + portion of body) at cytosines of the (A)** CG, **(B)** CHG and **(C)**, CHH contexts.

*Tos17* is an endogenous long terminal repeat (LTR) retrotransposon in the rice genome, and which is usually heavily methylated over it entire length under normal conditions. The methylation state of *Tos17* however is highly labile and loss of methylated cytosines (^m^Cs) may occur under various conditions, which may (but not necessarily) lead to its transpostional activation (Liu et al., 2004). Thus, *Tos17* has been widely accepted as a sensitive mark to assess methylation liability in rice (Liu et al., 2004; Ding et al., 2007). To verify that laser irradiation has caused changes in DNA methylation patterns and their transgenerational heritability by an independent approach, we analyzed methylation state of the 5′ portion of *Tos17* together with its immediate flank (Table 2) by locus-specific bisulfite sequencing in several M1 and M2 individuals that showed clear methylation changes in the MSAP profiles along with their WT. First, for the entire sequenced segment, there are two CG sites located in the flank region (Table 2), which are slightly methylated (*ca*. 10%) in WT, but the methylation was completely lost in all analyzed progeny individuals derived from the low-dose laser-treated mother plant (Figure 5A). There are nine CHG sites in the sequenced segment, four in the flank and five in the 5′ portion of *Tos17* (Table 2). In addition, there are 50 CHH sites in the sequenced segment, 27 in the flank and 23 in the body region of *Tos17* (Table 2). Analysis of these sequences found that (i) in WT, CG, CHG, and CHH sites were low-methylated, while only a small fraction of CHG sites had a high frequency of methylation. (ii) In the M1 plant, CG sites were slightly or moderately methylated compared with WT, most CHG sites were maintained the methylation patterns of M0, while CHH sites showed inheritance of M0 or reversal of the methylation patterns to those of WT. (iii) In the M2 plant, nearly all three kinds of methylation at CG, CHG and CHH were identical with those of M1 (Figure 5), denoting largely stable epigenetic inheritance thereafter.

**Table 2:**
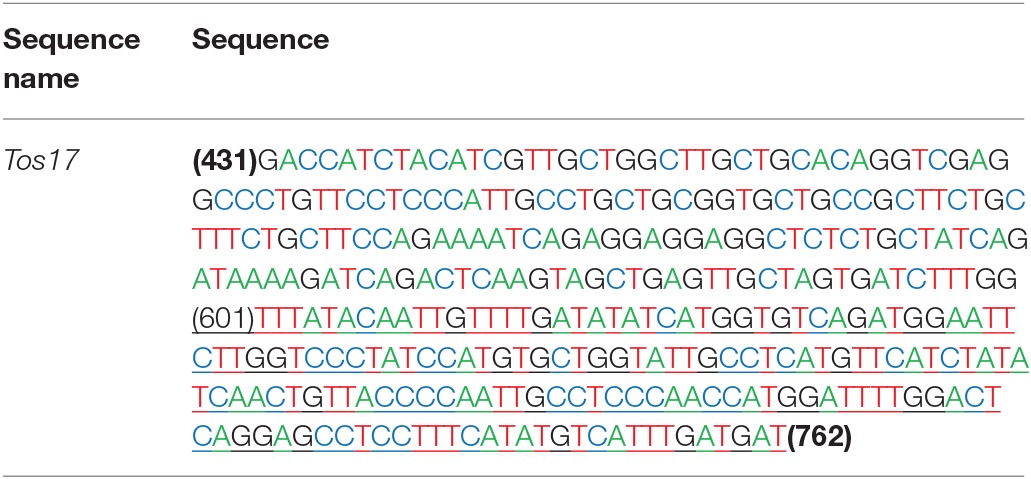
**The nucleotide sequence of the bisulfite sequenced region of *Tos17***.

*The analyzed region for Tos17 is 332 bp in length, with the first 170 bp being a portion of the 5′-LTR whereas the rest 162 bp being a portion of body-region (underlined)*.

### Transgenerational changes in expression of chromatin genes encoding enzymes directly related to DNA methylation

Establishment, erasure and maintenance of DNA methylation patterns are accomplished by the interlaced action of multiple enzymes encoded by a large set of genes collected termed chromatin genes (McCue et al., 2015; Rigal et al., 2016; Tan et al., 2016). To test whether the laser irradiation-induced heritable changes of DNA methylation patterns might be related to altered expression of the chromatin genes, especially those directly related to DNA methylation, we analyzed expression levels of a set of genes encoding DNA methyltransferases (*MET1-1, CMT3-1, DRM2-1*, and *DRM2-2*), 5-methylcytosine DNA glycosylases (*DME1, DME2*), the *SWI/SNF* chromatin remodeler (*DDM1*), and four siRNA pathway-related AGO proteins (*AGO1-1, AGO1-2, AGO4-1, AGO4-2*) in all the 11 M0 plants (Table 1). Real-time PCR results indicated that all the analyzed genes showed significantly perturbed expression in these M0 plants (Supplementary Table 6). Specifically, genes coding for DNA methyltransferases *CMT3-1, DRM2-1*, and *DRM2-2* were all significantly down-regulated in the M0 plants. Similarly, genes coding for 5-methylcytosine DNA glycosylases *DME1* and *DME2* were also down-regulated in the M0 plants, and so were genes coding for the siRNA pathway-related AGO proteins *AGO1-1, AGO1-2, AGO4-1*, and *AGO4-2* (Supplementary Table 6). In contrast, the gene coding for *SWI/SNF* chromatin remodeler *DDM1* was significantly up-regulated in all the M0 plants (Supplementary Table 6).

**Figure 6:**
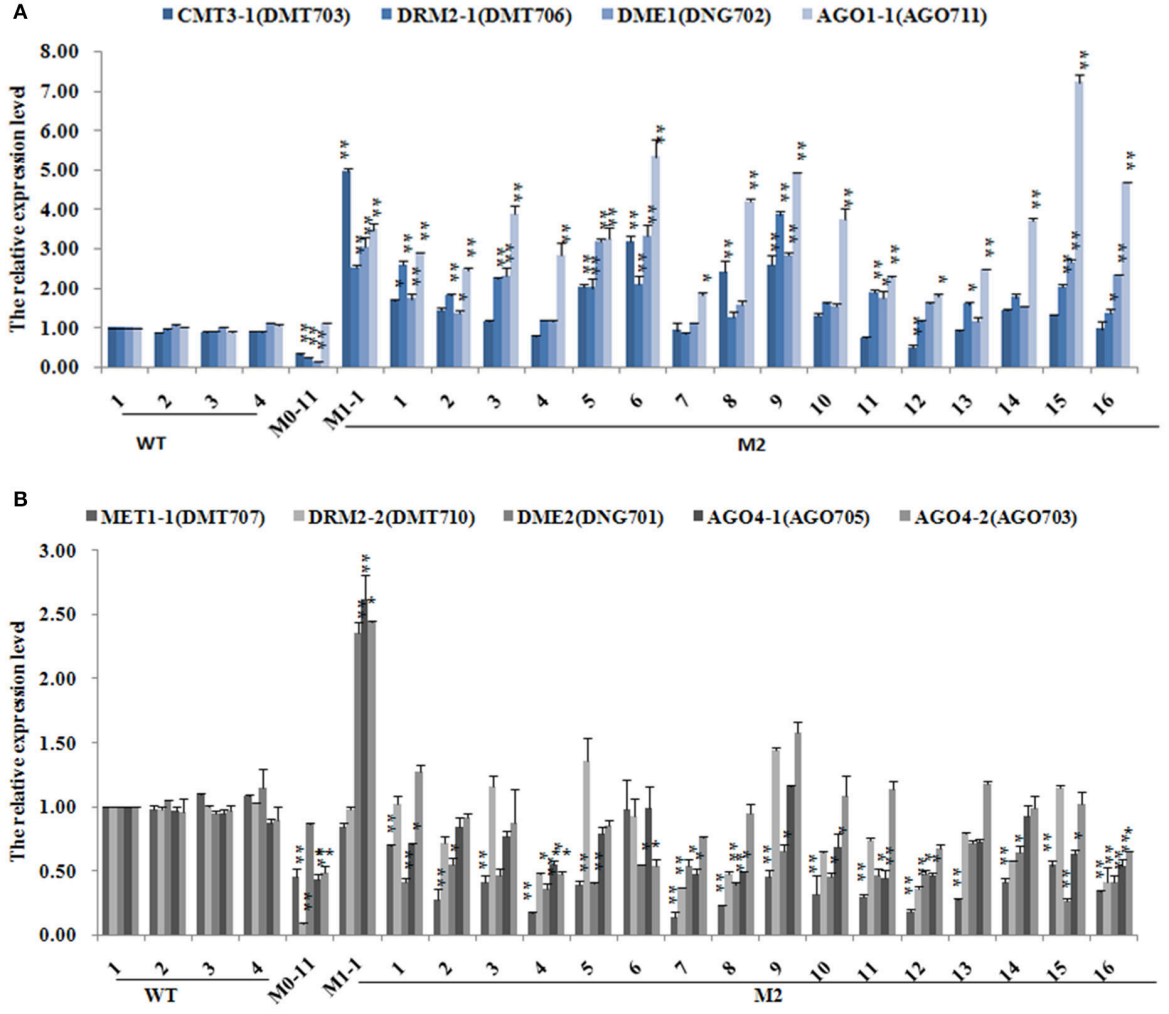
**The expression levels of different DNA methylation-related genes subsequent to the low-dose laser irradiation in M0, M1, M2 generation of Jinong 18. (A)** CMT3-1, DRM2-1, DME1, AGO1-1; **(B)** MET1-1, DRM2-2, DME2, AGO4-1, AGO4-2.

To test whether the significantly perturbed expression states of these chromatin genes in the M0 plants were also transgenerationally persistent, all the 18 M1 plants and 16 M2 plants were subjected to the same qRT-PCR analysis (Supplementary Table 7). Results indicated that *DDM1*, which was significantly up-regulated in M0, displayed a further up-regulation in virtually all the M1 plants, whereas *MET1-1, CMT3-1, DRM2-1, DME1, DME2, AGO1-1, AGO1-2, AGO4-1, AGO4-2*, which were down-regulated in M0 (Supplementary Table 6), showed up-regulated expression in nearly all the M1 plants (Supplementary Table 7). *DRM2-2*, which was also down-regulated in M0 (Supplementary Table 6), showed largely reversion to the expression state of WT (Supplementary Table 7). Notably, *DRM2-1* and *DRM2-2* showed a dramatic upregulation in two (M1-11 and M1-12) of the M1 plants (Supplementary Table 7). Further analysis of the M2 generation plants indicated that *CMT3, DRM2-1, DME1*, and *AGO1-1* showed stable inheritance of the expression states of their M1 mother plant (Figure 6A), while *MET1, DRM2-2, DME2, AGO4-1*, and *AGO4-2* showed largely reversion of expression states to those of the M0 plant, which nevertheless were still significantly different from those of WT (Figure 6B). *DDM1* which was significantly up-regulated in the M0 and M1 plants, showed a tendency of down-regulation in all but two (M2-5 and M2-6) of the studied M2 plants, and in these two plants it showed a highly outlier upregulation (Supplementary Table 8).

**Figure 7:**
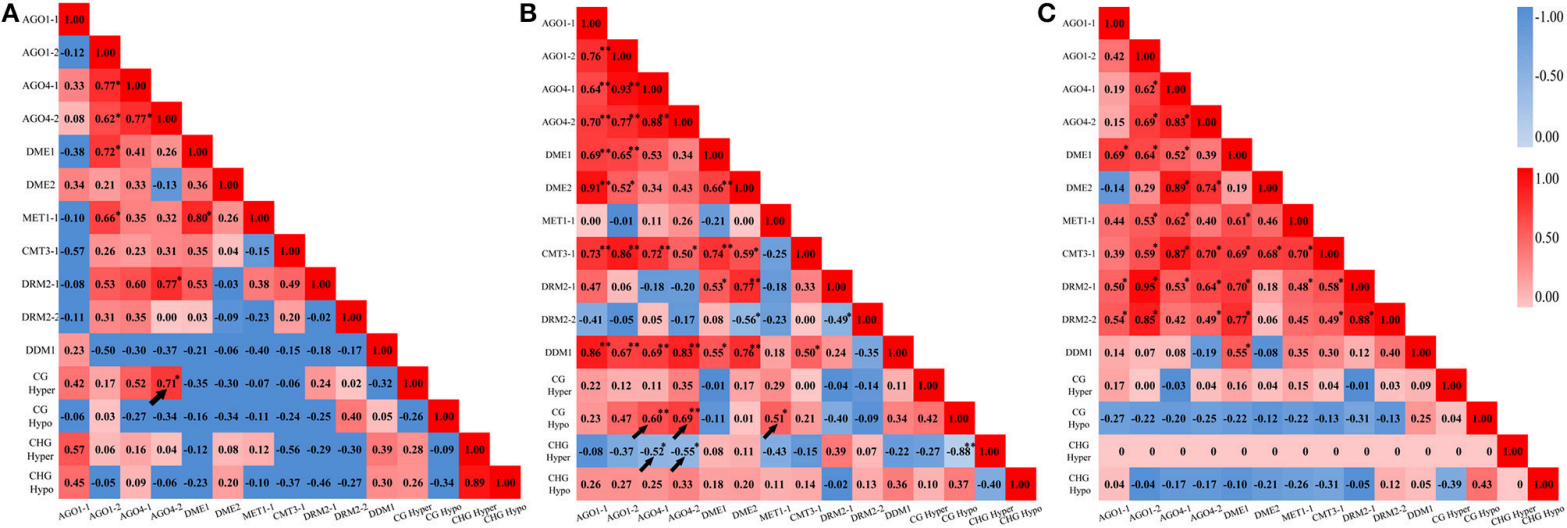
**Correlation analysis between cytosine methylation variations and the dynamic expression levels of the various genes related to DNA methylation**. **(A)** M0 generation; **(B)** M1 generation; **(C)** M2 generation.

In addition, A Pearson correlation analysis was used to analyze the possible correlations between the DNA methylation patterns (detected by MSAP) and the perturbed expression of chromatin genes (detected by qRT-PCR). The value of Pearson's r symbolizes the correlation tendency between the two variables. We found that, in M0 plants, CG hypermethylation was positively correlated with expression *AGO4-2* (*r* = 0.71, *P* < 0.05) (Figure 7A). In M1 plants, CG hypomethylation was positively correlated with expression of *AGO4-1* (*r* = 0.60, *P* < 0.01), *AGO4-2* (*r* = 0.69, *P* < 0.01) and *MET1-1* (*r* = 0.51, *P* < 0.05), while CHG hypermethylation was negatively correlated with expression *AGO4-1* (*r* = −0.52, *P* < 0.05) and *AGO4-2* (*r* = −0.55, *P* < 0.01) (Figure 7B). However, no correlation was detected in M2 plants, consistent with the MSAP profiles showing that the DNA methylation patterns were stabilized by large in the M2 generation (Figure 4C). Together, the above data of transgenerational expression dynamics suggested that the immediate changes, inheritance, additional changes, reversion to parental state as well as stabilization of the altered CG and CHG methylation patterns in the laser irradiated plants and their offspring are closely linked to the transgenerationally perturbed expression states of at least some of the chromatin genes analyzed.

It should be cautioned, however, that the expression dynamics of these chromatin-related genes both among the laser-irradiated individuals and between generations clearly did not follow a persistent trend. Therefore, we cannot rule out the possibility that stochastic factors like expression noise may have also contributed to the expression dynamics.

### Characterization of variant MASP loci in the laser-irradiated rice plants and progenies

To get some insights into the functional relevance of the genomic loci underwent methylation pattern changes as a result of laser-irradiation, 36 reproducible variant bands (shared among at least three M0 individuals) were eluted from the MSAP gel profile, cloned and sequenced. A BlastX query indicated that 16 loci were homologous to known or predicted protein-coding genes, three loci were TEs, and the other 15 loci showed no similarity with known sequences in the database (Supplementary Table 9), suggesting that at least certain variant methylation loci are likely functionally consequential.

### Transpositional activation of *mPing* in the laser-irradiated rice and its selfed progenies

**Figure 8:**
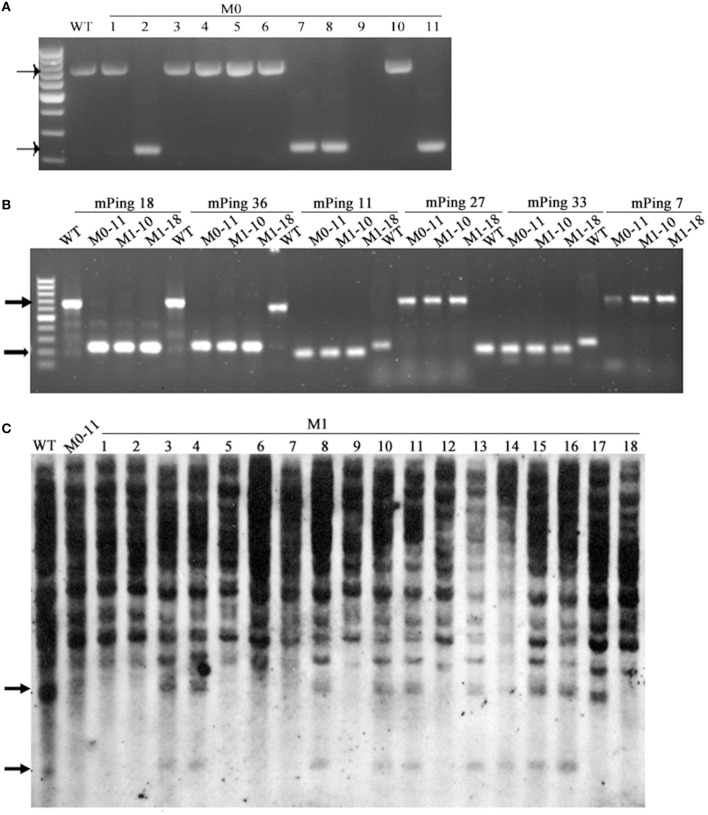
**The mobility of *mPing* induced by laser irradiation in Jinong 18. (A)** The *mPing* excisions detected in M0. **(B)** The *mPing* insertions and excisions detected in M1. **(C)** Southern blot analysis of the *mPing* mobility using separate probes.

*mPing* (430 bp in length) is the most active MITE-type TE in the rice genome, and can be induced to transpose under various stress conditions, and its mobility can be associated with DNA methylation of itself and/or its flanks (Ngezahayo et al., 2009). Given the extensive DNA methylation pattern changes induced by the laser irradiation, we sought to test the possibility that *mPing* had been mobilized by the treatment. For this purpose, 53 *mPing*-containing loci identified in the WT were analyzed by locus-specific PCR, and the results showed that *mPing* excision occurred in 21 (*ca*. 40%) loci in one or more of the 11 selected laser-irradiated M0 plants (Table 3), and *mPing* mobility was most active in M0#11 (Figure 8A). It has been documented previously that excision of *mPing* is not necessarily accompanied by reinsertions of the mobilized copies (Shan et al., 2005). To test if *mPing* reinsertions occurred in these plants, transposon-display (TD) was performed. Although exhaustive TD was conducted, only six reinsertion events in the M0#11 plant or some of its 19 M1 progenies (bands appeared *de novo* relative to WT), and all six loci were isolated and confirmed by newly designed PCR primers (e.g., Figure 8B). This suggests that although excisions of original *mPing* copies occurred abundantly (*ca*. 40%, 21 out of 53 loci), most mobilized copies failed to reinsert back to the rice genome. To confirm this possibility further, Southern blotting was conducted using the full-length *mPing* as a probe. Indeed, only loss of bands were detected in M0#11 and its progenies at the resolution of Southern blotting (Figure 8C), confirming the possibility, which is consistent with a previous study (Shan et al., 2005).

**Table 3:** **The statistical of dap sites of *mPing* in the M0 generation of Jinong18**.

**Primers**	**WT**	**1**	**2**	**3**	**4**	**5**	**6**	**7**	**8**	**9**	**10**	**11**
mPL2	+	−	−	−		−	−	+	−	−	+	−
mPL5	+	−	−	−		−	−	−	−	−	−	−
mPL7	+	+	+	+		+	+		+	+		+
mPL9	+	+	+	−		+	+	−	−	−	−	±
mPL10	+	+	+			+	+	+		+	+	+
mPL11	+	+	−	+	+	+	+	−	−		+	−
mPL12	+	−	−	−		−	−	−	−	−		−
mPL13	+	+	+	−		+	+	−	−	−	−	±
mPL16	+	−	−	−	−	−	−	−	−	−	−	−
mPL20	+	+	+	−	−	+	+	−	−	−	−	+
mPL21	+	−	−	−	−	−	−	−	−	−	−	−
mPL22	+	−	−	−	−	−	−	−	−	−	−	−
mPL23	+	−	−	−		+	+	+	+	+	−	−
mPL30	+	+	+	+		+	+		+	−	−	+
mPL31	+	+	+	−		+	+	−	−	−	−	±
mPL33	+	+	+	−		+	+					+
mPL37	+	−	−	−		−	−		−	−	−	−
mPL39	+	−	+	+		−	+		+	+		+
mPL42	+	−	−	−		−	+	−	−	−	−	−
mPL 45	+	−	−	+		−	−	+	+	+	+	−
mPL 48	+	−	−	−		−	−		−	−	−	−

*“+”represents existence of mPing at the locus (~700 bp),“−”represents absence of mPing at the locus and hence a lower band (~230 bp), space represents no bands amplified, “±” represents a locus with existence of mPing in a heterozygous state*.

## Discussion

Both in the evolutionary time-scale and during ontogenic lifecycle, plants encounter various internal and external stresses that may have negative impacts on their growth, development, reproduction and evolutionary success. These adverse conditions include recurring situations like abiotic and biotic stresses, as well as less frequent insults like interspecific hybridization and geological disasters. Conceivably, the strategies evolved in plants to cope with the conditions are diverse. Cong and Whitelaw have suggested that epigenetic modification is an efficiently evolved system that may enable rapid tuning by plants to enable adaptation to new environments (Youngson and Whitelaw, 2008). Previous researches showed that pathogen infection, nutrition or water deficiency, environmental pollution and tissue culture all induced DNA cytosine methylation variations (Kou et al., 2011; Ou et al., 2012; Gao et al., 2013; Wang et al., 2013; Zhang et al., 2014).

Low-dose laser irradiation has long been considered as a non-mutagenic treatment and widely used in agriculture to promote seed germination. Paradoxically, new crop cultivars were often selected from the treatments, which would entail the occurrence of heritable variations (Xu et al., 2008; Liu et al., 2016). Here we show that low-dose laser-irradiation could generate heritable phenotypic variants such as dwarfing and delayed development in rice in a dose-dependent manner. Apparently, either genetic mutation or heritable epigenetic changes or both must have occurred to these plants. Although we not rule out the possibility that the variant phenotypes were due to genetic mutations, given the well established notion, discussed above, that epigenetic mechanisms are readily responsive to all kinds of stressful conditions, and that the low-dose irradiation is certainly an unfamiliar condition that is not normally experienced by the rice plants, we suspect that the treatment that may have more likely instigated epigenetic instabilities. Indeed, we found extensive changes of DNA methylation patterns occurred in these phenotypically altered plants based on the MSAP analysis which in principle randomly samples at 5′CCGG sites across the genome. Importantly, we document that a significant proportion of the altered methylation patterns is inherited to organismal progenies of the laser-irradiated plants, and forming new epigenetic alleles. This kind of epigenetic variation and inheritance of unusual parental experience is known as evolutionarily consequential (Jablonka and Lamb, 2015). By contrast, in the MSAP profiles we did not observe identical variant bands that appeared in digests by the two isoschizomers (*Hpa*II and *Msp*I), suggesting that if nucleotide sequence mutations were generated by the low-dose laser treatments, they were insignificant compared with changes in DNA methylation.

We show that chromatin-related genes encoding DNA methyl-transferase CMT3, the *de novo* methylase DRM2-1, DNA glycosylase DME1 and the siRNA-related proteins AGO1-1 were down-regulated immediately after the laser-irradiation in the M0 plants, but they were up-regulated in the M1 plants, and which was then followed by relatively stable inheritance of the elevated expression state in the M2 plants (Figure 6A). Similarly, genes encoding DNA methyl-transferase MET1, the *de novo* methylase DRM2-2 and the siRNA-related proteins AGO4-1 and AGO4-2 were down-regulated after laser-irradiation in M0, and up-regulated in M1, but then largely reversed to the expression levels of M0 in M2 (Figure 6B). These observations suggest that the heritable changes of DNA methylation patterns is likely due to transgenerationally perturbed expression of these chromatin-regulation genes by the low-dose laser irradiation. Conceivably, with time, the expression of these genes will be reversed to the original default states (i.e., in the WT), but the newly acquired methylation patterns will be perpetuated, and thus different from that of the WT. Indeed, a correlation analysis indicated that at least some of the methylation pattern changes are significantly correlated with expression dynamics of some of the chromatin genes. If the heritable changes of DNA methylation bear functional impacts, then some of their readout phenotypes, if adaptive, can be fixed under natural settings or selected artificially for breeding purposes. The fact that at least some of the variant MSAP loci are associated with known or predicted protein-coding genes lent support to this possibility.

TEs account for 35% of the rice genome. Although the great majority of TEs have become defective due to sequence changes (e.g., truncation or nested insertion by other TEs), a small fraction of TEs, being primarily repressed by epigenetic mechanisms, are largely intact at the sequence level and potentially active. Indeed, the majority of DNA methylation was found to map to TEs in the rice genome (Law et al., 2010). In this respect, we note that the bisulfite results mainly showing hypermethylation at cytosines of all sequence contexts (CG, CHG, and CHH) in virtually all the analyzed *Tos17* region in progenies of the laser-treated plant, which is consistent with the consistently elevated transcript abundance of DDM1 that is known as required to establish and perpetuate cytosine methylation at TEs of all sequence contexts (Saze and Kakutani, 2007; Ito et al., 2015). Therefore, its significant and persistent upregulation may have contributed to the increased methylation of TEs like *Tos17*. Notably, under various internal and external stress conditions, the normally dormant TEs can be re-weaken and become mobilized (McClintock, 1984), and which often is accompanied by disruption of repressive epigenetic marks, e.g., in the case of *mPing* (Ngezahayo et al., 2009). Specifically, it was documented that the transpositional activity of *mPing* was closely correlated altered methylation states both of itself and its upstream flanks under tissue culture conditions (Ngezahayo et al., 2009). We show here that *mPing* is also mobilized in the laser-irradiated rice plants which showed alterations in DNA methylation and phenotypic variations. It is therefore likely that mobilization of *mPing* by the treatment is also correlated with DNA methylation changes. An interesting observation is that great majority of the excised *mPing* copies were not re-integrated into the rice genome, consistent with an earlier report in other situations (Shan et al., 2009). Still the excisions alone may have phenotypic consequences because *mPing* contains regulatory sequences which impact regulation of genes residing at its flanks under various stress conditions (Naito et al., 2009; Ito et al., 2016). It will be interesting to test if the progeny plants derived from the laser-irradiated mother plants, which have lost many original copies of *mPing*, will respond differently to stress conditions.

## Author contributions

SL, QX, and XY performed most of the experiments. JM and HK contributed to seedling planting, sample preparation and phenotyping, XL contributed to data collection. XG and BL coordinated the project, and conceived the study. FW, XG, and BL analyzed the data and wrote the manuscript. All authors read and approved the final manuscript.

## Funding

This work was supported by the State Key Basic Research and Development Plan of China (2013CBA01404) and the Program for Introducing Talents to Universities (B07017). The funders had no role in study design, data collection and analysis, decision to publish, or preparation of the manuscript.

### Conflict of interest statement

The authors declare that the research was conducted in the absence of any commercial or financial relationships that could be construed as a potential conflict of interest.
