# Supplementary material for: Laser Irradiation-Induced DNA Methylation Changes Are Heritable and Accompanied with Transpositional Activation of mPing in Rice
Source: Front Plant Sci. 2017 Mar 21;8:363. doi: 10.3389/fpls.2017.00363 (PMC5359294; doi:10.3389/fpls.2017.00363)
Supplement: Supplementary file 1 [file Table1.DOCX]

Supplementary Table1. Adapters, pre-amplification and selective amplification primers of MSAP

| primers | Sequence (5’-3’) |
| --- | --- |
| **Adapters** | |
| *EcoRI*-adapterI | CTCGTAGACTGCGTACC |
| *EcoRI*-adapterlI | AATTGGTACGCAGTC |
| *H/M*-adapterI | GATCATGAGTCCTGCT |
| *H/M*—adapterlI | CGAGCAGGACTCATGA |
| **Pre-selective primers** | |
| *EcoRI*+A | GACTGCGTACCAATTCA |
| H/M+0 | ATCATGAGTCCTGCTCGG |
| **EcoRI primers** | |
| E-AAC | GACTGCGTACCAATTCAAC |
| E-AAG | GACTGCGTACCAATTCAAG |
| E-ACA | GACTGCGTACCAATTCACA |
| E-ACT | GACTGCGTACCAATTCACT |
| E-ACC | GACTGCGTACCAATTCACC |
| E-ACG | GACTGCGTACCAATTCACG |
| E-AGC | GACTGCGTACCAATTCAGC |
| E-AGG | GACTGCGTACCAATTCAGG |
| E-AGA | GACTGCGTACCAATTCAGA |
| E-ATC | GACTGCGTACCAATTrATC |
| **H/M primers** | |
| H/M-TCT | ATCATGAGTCCTGCTCGGTCT |
| H/M-TCG | ATCATGAGTCCTGCTCGGTCG |
| H/M-TCC | ATCATGAGTCCTGCTCGGTCC |
| H/M-TTC | ATCATGAGTCCTGCTCGGTTC |
| H/M-TTG | ATCATGAGTCCTGCTCGGTTG |
| H/M-TTA | ATCATGAGTCCTGCTCGGTTA |
| H/M-TGA | ATCATGAGTCCTGCTCGGTGA |
| H/M-TGT | ATCATGAGTCCTGCTCGGTGT |
| H/M-TGC | ATCATGAGTCCTGCTCGGTGC |
| H/M-TAC | ATCATGAGTCCTGCTCGGTAC |
