# Supplementary material for: Laser Irradiation-Induced DNA Methylation Changes Are Heritable and Accompanied with Transpositional Activation of mPing in Rice
Source: Front Plant Sci. 2017 Mar 21;8:363. doi: 10.3389/fpls.2017.00363 (PMC5359294; doi:10.3389/fpls.2017.00363)
Supplement: Supplementary file 2 [file Table2.docx]

Supplementary Table 2. Primers used in qRT-PCR

|  | Genes | ChromDB ID | Primer sequences (3’) | |
| --- | --- | --- | --- | --- |
|  |  |  | Forward | Reverse |
| Methyltransferases | METI-1 | DMT707 | CAGGTGGATTCAGATAAATGTA | AATCGCCCACTTTGTAAACG |
|  | CMT3-1 | DMT703 | GGTTTCGTGGAACAGTCAA | GGCGGCAGCAACATAAGG |
|  | DRM2-1 | DMT706 | CCAAGGAGGCAGTTCAA | CATTCGTCCAAGACATAC |
|  | DRM2-2 | DMT710 | ACACCAGGCATCCGTAG | ATGTAACCCTCCTTTCG |
|  | DDM1 | CHR741 | GGGCTGGTGGGCTGGGTAT | GCATTAGGTTTGGCTCTGTCT |
| Methylcytosine DNA glycosylases | DME1 | DNG702 | ACTGGAAACGCTGTCG | CTGCTGCCAATGTCTT |
|  | DME2 | DNG701 | CACTGATTTCAACAGGGACA | AATAAGATACACCAAAGCC |
| siRNA-related proteins | AGO1-1 | AG0711 | TGGGAATGTTGCTGTAAGG | GGCGACTCAAACCGTCAGAC |
|  | AGO1-2 | AG0708 | GACCAGCCAAGCCATTCA | TCAAACATAACAGCACGATT |
|  | AGO4-1 | AG0705 | CTGATCCCATGTTGAGGGC | TTGAAGTTCCACCGACCA |
|  | AGO4-2 | AG0703 | TCCAAGGTCCCGACAATA | TCAACTAATAACTCCCGAATG |
| Internal control | β-actin |  | ATGCCATTCTCCGTCTT | GCTCCTGCTCGTAGTC |
