# Supplementary material for: Laser Irradiation-Induced DNA Methylation Changes Are Heritable and Accompanied with Transpositional Activation of mPing in Rice
Source: Front Plant Sci. 2017 Mar 21;8:363. doi: 10.3389/fpls.2017.00363 (PMC5359294; doi:10.3389/fpls.2017.00363)
Supplement: Supplementary file 3 [file Table3.DOCX]

Supplementary Table 3. List of 53 *mPing*-containing primers designed based on the whole genome sequence of Nipponbare ((<http://rgp.dna.affrc.go.jp>).

| Locus | Chromosome | Primers | |
| --- | --- | --- | --- |
|  |  | Forward (5'-3') | Reverse (5'-3') |
| MpL10 | 1 | gaaactaacgcgtgcacaga | gcgattcagcataacaccaa |
| MpL15 | 1 | tggctggtccttaccttttg | gacgtggagaggtggaagag |
| MpL29 | 1 | cgaatgcatcgataccactta | taatggcccaattcaatgct |
| MpL39 | 1 | tgtggttgtggtagctgcat | ctgtaccgcacggcagtatt |
| MpL3 | 2 | ggggagttgcaagtgttgat | tcctcaaaacagccatagca |
| MpL5 | 2 | gcagccagtacgtagcacag | acgaacgtgggctgttttag |
| MpL14 | 2 | tgagcgagagaaattaatccaa | tccttgtcctctcaaatgcac |
| MpL30 | 2 | aagcccgggataagagattc | caacggatgatcagatgtgc |
| MpL34 | 2 | aaacccacggtttgcttttt | ggaagacagagccactgagc |
| MpL38 | 2 | caaagccaaaacaaggatgc | aagggcgcatattagcaaaa |
| MpL40 | 2 | tatctgagcgtgagcgtgtc | ttatttggggacgacctttg |
| MpL47 | 2 | atcgccttaacaccgtcata | ccctcactccggttgaatac |
| MpL7 | 3 | gctcgtggctgaagacctta | gctcgtggctgaagacctta |
| MpL11 | 3 | tcccattcaaagatgacgaa | gaacacgaaacaacagaacacc |
| MpL13 | 3 | atagtcaggattccggcaaa | ttcgagaacgattgctgttg |
| MpL23 | 3 | ttgagagcatccacaacgaa | atcggcattagcacaaagga |
| MpL24 | 3 | tttgcctttctgctgatcct | aacgatgccaaagtatgctg |
| MpL26 | 3 | ggagatctgagcgagtttgc | gggaacaaaagctaggagca |
| MpL27 | 3 | gcaggcagatgttgatggta | tttgcatgcttgcttggtat |
| MpL28 | 3 | agcgatggtgcattggttat | ggaagctgctgcttttgaag |
| MpL36 | 3 | catgtgcgtggaaaacagag | ggtgcggaacatgtcatcta |
| MpL53 | 3 | tttacgtcaggggaatggac | tccgcgttcttcagtttcta |
| MpL4 | 4 | tggtttgctgggacatgtaa | gctcttgcataagagccaaca |
| MpL20 | 4 | ggcaatggtgattcgttga | tgcatgagagccaatactcc |
| MpL22 | 4 | ctgcacgcctagcctcttta | agcgctcgactactccagat |
| MpL33 | 4 | gtggagaaaatgggtgagga | tacgggtgttgacatgaagc |
| MpL41 | 4 | acaatcaatggcttccttgc | ccaagtgtcatgcctgctta |
| MpL6 | 5 | tttgtcggcgtctactccat | tttgcagctggcttatagca |
| MpL35 | 5 | atgcaaagatttggtgagca | cccacacctttgatttttcg |
| MpL43 | 5 | ccaaatgagcccgtaaatct | tccagactccagttctgcac |
| MpL31 | 6 | acagcaatcaccacacgaaa | ctgagatggaatcggcaaat |
| MpL44 | 6 | gtccgatggatcctactggt | attaagcatgcatgggtgtg |
| MpL46 | 6 | tacggagagcattgtgttcc | cttgttcaatccacgtcctg |
| MpL48 | 6 | gaggcaggagattagggttg | gacaatgcccactgttagga |
| MpL18 | 7 | gcacaggctccaagacgta | aaaaactgaccgttggatgg |
| MpL9 | 8 | cggagcacggagtacttatca | gctctaaatcacctagccaacg |
| MpL16 | 8 | gggctccacgtcatcataa | tcagctccaccaaaactgg |
| MpL17 | 8 | atctccatcccctcacgac | aaaagtgtcggaagctctgc |
| MpL37 | 8 | tgaggcattgaggtgcacta | cgctatattaatgccggttcc |
| MpL45 | 8 | tcctcctactcctccacagc | cacaacaggcaacctcaact |
| MpL50 | 8 | aaagagaaaagcagcggact | aaatgacggttttgttttgc |
| MpL1 | 9 | aggcgcaatcattagtacgg | catcggattctcctcccatc |
| MpL51 | 9 | ccaaccgttctggttcacta | gagtcacgtggaccgaatag |
| MpL25 | 10 | ttccttcggacaggcttatg | cgaacttcctgtctctgcact |
| MpL42 | 10 | gtgggaagtgatgaggagga | cgcgggggattagaatactt |
| MpL2 | 11 | ggatgctttgttagcgcaat | catccttctcctccaactcc |
| MpL12 | 11 | tactgccttttgctccatcc | caggctttgccaatagaaca |
| MpL52 | 11 | gccgcgagctaatgatagtt | gtaaccctgccctgactcat |
| MpL8 | 12 | atgtgcactgtgcctggtag | tctcgctctttcagtgagca |
| MpL19 | 12 | cccggaatcatggctactt | ttctcggatcaccaaagacc |
| MpL21 | 12 | cccatttgaataccggatga | ctgggcaacttggagtacg |
| MpL32 | 12 | tcaagaacagtgccaactcg | catacgccctattccgttgt |
| MpL49 | 12 | aatcgcgaaaatgaactctg | ggcacagctcctaacaggta |
