# Supplementary material for: Laser Irradiation-Induced DNA Methylation Changes Are Heritable and Accompanied with Transpositional Activation of mPing in Rice
Source: Front Plant Sci. 2017 Mar 21;8:363. doi: 10.3389/fpls.2017.00363 (PMC5359294; doi:10.3389/fpls.2017.00363)
Supplement: Supplementary file 4 [file Table4.DOCX]

Supplementary Table 4. Adapters, pre-amplification primers, *mPing* internal amplification primers, *mPing* proximal ends amplification primers and selective primers of TD

| primer | sequence |
| --- | --- |
| MseI Adapter | 5’- GACGATGAGTCCTGAG |
| MseI＋0 pre amplification primer | 5’- GATGAGTCCTGAGTAA |
| *mPing* internal amplification primers | 5’- GCTGACGAGTTTCACCAGGATG |
| *mPing* proximal ends amplification primers | 5’- TGTGCATGACACACCAGTG |
| MseI＋2 selective amplification primers | |
| M-CAA | 5’-GATGAGTCCTGAGTAACAA |
| M-CAC | 5’-GATGAGTCCTGAGTAACAC |
| M-CAG | 5’-GATGAGTCCTGAGTAACAG |
| M-CAT | 5’-GATGAGTCCTGAGTAACAT |
| M-CTA | 5’-GATGAGTCCTGAGTAACTA |
| M-CTC | 5’-GATGAGTCCTGAGTAACTC |
| M-CTG | 5’-GATGAGTCCTGAGTAACTG |
| M-CTT | 5’-GATGAGTCCTGAGTAACTT |
