# Supplementary material for: Laser Irradiation-Induced DNA Methylation Changes Are Heritable and Accompanied with Transpositional Activation of mPing in Rice
Source: Front Plant Sci. 2017 Mar 21;8:363. doi: 10.3389/fpls.2017.00363 (PMC5359294; doi:10.3389/fpls.2017.00363)
Supplement: Supplementary file 5 [file Table5.DOCX]

Supplementary Table 5. The primers used for Bisulfite sequencing

| primers | sequence | size |
| --- | --- | --- |
| Tos17-BS-F1 | 5' –TGTGYATAGGATAYATTYTCGTTGAA- 3' | 535 bp |
| Tos17-BS-R1 | 5' –ATAAATATRAATTRRARRARRTTRCTTA-3' |  |
| Tos17-BS-F2 | 5' –YATGTGTGGTTTYTATYAYTYGGATGT-3' | 332 bp |
| Tos17-BS-R2 | 5' –RTTCARACCATTRCTCTRATACCATCT-3' |  |
