# Supplementary material for: Laser Irradiation-Induced DNA Methylation Changes Are Heritable and Accompanied with Transpositional Activation of mPing in Rice
Source: Front Plant Sci. 2017 Mar 21;8:363. doi: 10.3389/fpls.2017.00363 (PMC5359294; doi:10.3389/fpls.2017.00363)
Supplement: Supplementary file 6 [file Table6.DOC]

Supplementary Table 6. The relative expression of chromatin related genes in M0 progeny

|  | MET1-1  (DMT707) | CMT3-1  (DMT703) | DRM2-1  (DMT706) | DRM2-2  (DMT710) | DDM1  (CHR741) | DME1  (DNG702) | DME2  (DNG701) | AGO1-1  (AGO711) | AGO1-2  (AGO708) | AGO4-1  (AGO705) | AGO4-2  (AGO703) |
| --- | --- | --- | --- | --- | --- | --- | --- | --- | --- | --- | --- |
| WT1 | 1.00+0.00 | 1.00+0.00 | 1.00+0.00 | 1.00+0.00 | 1.00+0.00 | 1.00+0.00 | 1.00+0.00 | 1.00+0.00 | 1.00+0.00 | 1.00+0.00 | 1.00+0.00 |
| WT2 | 0.97+0.03 | 0.87+0.01 | 0.95+0.04 | 0.98+0.02 | 0.97+0.00 | 1.06+0.03 | 1.05+0.00 | 1.02+0.01 | 0.95+0.07 | 0.97+0.04 | 0.96+0.10 |
| WT3 | 1.09+0.01 | 0.88+0.03 | 0.91+0.01 | 1.00+0.01 | 0.95+0.03 | 1.00+0.00 | 0.94+0.02 | 0.87+0.06 | 0.95+0.05 | 0.95+0.03 | 0.97+0.05 |
| WT4 | 1.08+0.01 | 0.90+0.02 | 0.88+0.02 | 1.03+0.00 | 0.96+0.04 | 1.12+0.01 | 1.15+0.15 | 1.05+0.05 | 0.97+0.10 | 0.87+0.03 | 0.89+0.11 |
| **MEAN(WT)** | **1.04+0.01** | **0.91+0.01** | **0.94+0.02** | **1.00+0.01** | **0.97+0.02** | **1.05+0.01** | **1.04+0.04** | **0.98+0.03** | **0.97+0.05** | **0.95+0.02** | **0.95+0.06** |
| M0-1 | 0.92+0.01 | 0.41+0.03 | 0.36+0.03 | 0.42+0.01 | 2.60+0.09 | 0.66+0.07 | 0.68+0.01 | 0.71+0.02 | 0.56+0.05 | 0.73+0.08 | 0.58+0.07 |
| M0-2 | 0.73+0.07 | 0.49+0.05 | 0.45+0.00 | 0.44+0.00 | 1.92+0.17 | 0.62+0.05 | 0.99+0.13 | 0.95+0.08 | 0.70+0.07 | 1.67+0.14 | 0.95+0.07 |
| M0-3 | 1.01+0.18 | 0.31+0.09 | 0.38+0.01 | 0.29+0.06 | 2.63+0.44 | 0.47+0.03 | 0.63+0.05 | 1.16+0.08 | 0.52+0.02 | 1.35+0.12 | 0.91+0.13 |
| M0-4 | 0.84+0.21 | 0.46+0.04 | 0.44+0.00 | 0.12+0.04 | 2.90+0.09 | 0.65+0.02 | 0.61+0.04 | 0.65+0.01 | 0.35+0.02 | 0.63+0.03 | 0.72+0.02 |
| M0-5 | 1.01+0.23 | 0.39+0.01 | 0.23+0.03 | 0.31+0.02 | 2.10+0.06 | 0.65+0.02 | 0.86+0.11 | 0.67+0.01 | 0.44+0.02 | 0.72+0.06 | 0.52+0.06 |
| M0-6 | 1.76+0.56 | 0.38+0.01 | 0.42+0.00 | 0.06+0.01 | 1.66+0.21 | 0.96+0.04 | 0.98+0.07 | 0.89+0.01 | 0.95+0.06 | 1.36+0.00 | 0.91+0.02 |
| M0-7 | 1.49+0.14 | 0.36+0.01 | 0.42+0.01 | 0.12+0.02 | 1.90+0.04 | 0.80+0.03 | 0.98+0.05 | 0.93+0.01 | 0.42+0.01 | 0.93+0.00 | 0.68+0.04 |
| M0-8 | 0.96+0.04 | 0.34+0.01 | 0.35+0.01 | 0.04+0.00 | 3.91+0.07 | 0.59+0.01 | 0.90+0.04 | 1.01+0.07 | 0.31+0.00 | 0.87+0.05 | 0.70+0.01 |
| M0-9 | 0.88+0.09 | 0.43+0.13 | 0.35+0.06 | 0.25+0.00 | 3.54+0.55 | 0.77+0.07 | 1.10+0.05 | 1.04+0.01 | 0.45+0.02 | 1.05+0.03 | 0.50+0.07 |
| M0-10 | 0.80+0.08 | 0.53+0.03 | 0.41+0.03 | 0.07+0.01 | 2.35+0.05 | 0.59+0.08 | 0.78+0.01 | 0.75+0.00 | 0.40+0.03 | 0.90+0.07 | 0.85+0.01 |
| M0-11 | 0.45+0.07 | 0.30+0.04 | 0.23+0.02 | 0.08+0.01 | 2.51+0.38 | 0.14+0.01 | 0.86+0.01 | 1.11+0.01 | 0.10+0.01 | 0.44+0.04 | 0.48+0.06 |
| **MEAN**  **(M0)** | **0.99+0.15** | **0.40+0.04** | **0.37+0.02** | **0.20+0.02** | **2.55+0.20** | **0.63+0.04** | **0.85+0.05** | **0.90+0.03** | **0.47+0.03** | **0.97+0.06** | **0.71+0.05** |
