# Supplementary material for: Laser Irradiation-Induced DNA Methylation Changes Are Heritable and Accompanied with Transpositional Activation of mPing in Rice
Source: Front Plant Sci. 2017 Mar 21;8:363. doi: 10.3389/fpls.2017.00363 (PMC5359294; doi:10.3389/fpls.2017.00363)
Supplement: Supplementary file 7 [file Table7.DOC]

Supplementary Table7. The relative expression of chromatin related genes in M1 progeny

|  | MET1-1  (DMT707) | CMT3-1  (DMT703) | DRM2-1  (DMT706) | DRM2-2  (DMT710) | DDM1  (CHR741) | DME1  (DNG702) | DME2  (DNG701) | AGO1-1  (AGO711) | AGO1-2  (AGO708) | AGO4-1  (AGO705) | AGO4-2  (AGO703) |
| --- | --- | --- | --- | --- | --- | --- | --- | --- | --- | --- | --- |
| **MEAN(WT)** | **1.04+0.01** | **0.91+0.01** | **0.94+0.02** | **1.00+0.01** | **0.97+0.02** | **1.05+0.01** | **1.04+0.04** | **0.98+0.03** | **0.97+0.05** | **0.95+0.02** | **0.95+0.06** |
| **MEAN(M0)** | **0.99+0.15** | **0.40+0.04** | **0.37+0.02** | **0.20+0.02** | **2.55+0.20** | **0.63+0.04** | **0.85+0.05** | **0.90+0.03** | **0.47+0.03** | **0.97+0.06** | **0.71+0.05** |
| M1-1 | 0.84+0.03 | 4.97+0.05 | 2.53+0.06 | 0.98+0.02 | 2.50+0.00 | 3.04+0.24 | 2.36+0.08 | 3.48+0.15 | 2.85+0.06 | 2.62+0.19 | 2.44+0.01 |
| M1-2 | 1.58+0.02 | 1.52+0.05 | 2.15+0.06 | 2.10+0.04 | 3.05+0.07 | 2.29+0.07 | 1.98+0.00 | 2.40+0.07 | 1.57+0.10 | 1.41+0.01 | 2.52+0.10 |
| M1-3 | 1.29+0.09 | 0.10+0.01 | 0.07+0.00 | 0.99+0.00 | 0.13+0.00 | 0.10+0.00 | 0.09+0.00 | 0.10+0.00 | 0.06+0.00 | 0.06+0.00 | 0.11+0.00 |
| M1-4 | 2.03+0.01 | 1.83+0.08 | 1.77+0.03 | 1.08+0.00 | 3.74+0.13 | 2.95+0.23 | 2.08+0.08 | 2.45+0.14 | 1.94+0.04 | 1.99+0.19 | 2.34+0.02 |
| M1-5 | 2.88+0.16 | 0.97+0.05 | 0.95+0.03 | 1.01+0.00 | 1.14+0.20 | 1.57+0.04 | 1.01+0.03 | 1.11+0.19 | 1.03+0.02 | 1.06+0.06 | 1.38+0.01 |
| M1-6 | 2.98+0.01 | 1.64+0.04 | 1.79+0.01 | 1.42+0.00 | 4.01+0.18 | 2.10+0.12 | 2.04+0.15 | 2.09+0.10 | 1.30+0.08 | 1.70+0.15 | 2.08+0.02 |
| M1-7 | 1.11+0.01 | 0.34+0.00 | 0.50+0.01 | 1.05+0.00 | 0.82+0.07 | 0.37+0.00 | 0.44+0.01 | 0.47+0.05 | 0.46+0.02 | 0.42+0.02 | 0.54+0.02 |
| M1-8 | 1.02+0.01 | 0.75+0.03 | 0.88+0.00 | 1.04+0.00 | 1.28+0.03 | 0.82+0.07 | 0.98+0.09 | 0.90+0.14 | 0.74+0.01 | 0.81+0.03 | 0.95+0.05 |
| M1-9 | 0.73+0.04 | 2.15+0.04 | 1.95+0.05 | 1.38+0.05 | 4.30+0.67 | 1.79+0.11 | 2.37+0.05 | 2.83+0.17 | 1.32+0.02 | 1.46+0.03 | 2.33+0.10 |
| M1-10 | 1.55+0.20 | 2.15+0.09 | 2.17+0.01 | 1.03+0.00 | 5.19+0.13 | 3.39+0.41 | 3.51+0.24 | 4.19+0.14 | 1.86+0.16 | 1.60+0.08 | 2.42+0.08 |
| M1-11 | 0.97+0.11 | 2.97+0.07 | 47.29+0.93 | 29.48+0.56 | 3.35+0.34 | 3.65+0.05 | 4.06+0.30 | 3.77+0.30 | 1.34+0.26 | 0.83+0.06 | 0.82+0.00 |
| M1-12 | 0.90+0.12 | 2.76+0.24 | 52.02+2.55 | 33.84+1.42 | 4.23+0.48 | 4.11+0.38 | 4.69+0.14 | 4.03+0.13 | 1.31+0.04 | 0.87+0.08 | 1.18+0.16 |
| M1-13 | 0.56+0.05 | 0.37+0.02 | 0.35+0.02 | 0.98+0.02 | 0.61+0.01 | 0.68+0.00 | 0.60+0.04 | 0.69+0.02 | 0.31+0.00 | 0.46+0.05 | 0.52+0.02 |
| M1-14 | 1.55+0.00 | 1.28+0.17 | 1.10+0.02 | 1.15+0.01 | 1.95+0.05 | 2.15+0.07 | 1.36+0.02 | 2.00+0.24 | 0.96+0.01 | 1.44+0.06 | 1.88+0.09 |
| M1-15 | 0.93+0.03 | 2.88+0.07 | 3.48+0.05 | 2.79+0.09 | 6.16+0.24 | 2.79+0.04 | 3.06+0.03 | 4.11+0.33 | 2.55+0.01 | 2.79+0.39 | 3.18+0.17 |
| M1-16 | 1.86+0.04 | 1.89+0.10 | 2.30+0.02 | 2.10+0.00 | 2.90+0.14 | 2.63+0.05 | 1.84+0.11 | 2.54+0.09 | 2.27+0.16 | 1.96+0.01 | 1.85+0.05 |
| M1-17 | 0.07+0.05 | 2.68+0.20 | 1.80+0.00 | 2.09+0.04 | 1.07+0.06 | 4.62+0.05 | 0.43+0.03 | 0.95+0.04 | 1.65+0.23 | 1.58+0.12 | 0.72+0.07 |
| M1-18 | 0.68+0.12 | 0.76+0.03 | 0.94+0.01 | 1.02+0.00 | 1.54+0.13 | 1.56+0.14 | 0.95+0.08 | 0.82+0.03 | 0.84+0.03 | 0.75+0.11 | 0.92+0.03 |
| **MEAN(M1)** | **1.31+0.06** | **1.78+0.07** | **6.89+0.21** | **4.75+0.13** | **2.66+0.16** | **2.25+0.12** | **1.88+0.08** | **2.16+0.13** | **1.35+0.07** | **1.32+0.09** | **1.57+0.06** |
