# Supplementary material for: Laser Irradiation-Induced DNA Methylation Changes Are Heritable and Accompanied with Transpositional Activation of mPing in Rice
Source: Front Plant Sci. 2017 Mar 21;8:363. doi: 10.3389/fpls.2017.00363 (PMC5359294; doi:10.3389/fpls.2017.00363)
Supplement: Supplementary file 8 [file Table8.DOC]

Supplementary Table 8. The relative expression of chromatin related genes in M2 progeny

|  | MET1-1  (DMT707) | CMT3-1  (DMT703) | DRM2-1  (DMT706) | DRM2-2  (DMT710) | DDM1  (CHR741) | DME1  (DNG702) | DME2  (DNG701) | AGO1-1  (AGO711) | AGO1-2  (AGO708) | AGO4-1  (AGO705) | AGO4-2  (AGO703) |
| --- | --- | --- | --- | --- | --- | --- | --- | --- | --- | --- | --- |
| **MEAN(WT)** | **1.04+0.01** | **0.91+0.01** | **0.94+0.02** | **1.00+0.01** | **0.97+0.02** | **1.05+0.01** | **1.04+0.04** | **0.98+0.03** | **0.97+0.05** | **0.95+0.02** | **0.95+0.06** |
| **MEAN(M0)** | **0.99+0.15** | **0.40+0.04** | **0.37+0.02** | **0.20+0.02** | **2.55+0.20** | **0.63+0.04** | **0.85+0.05** | **0.90+0.03** | **0.47+0.03** | **0.97+0.06** | **0.71+0.05** |
| **MEAN(M1)** | **1.31+0.06** | **1.78+0.07** | **6.89+0.21** | **4.75+0.13** | **2.66+0.16** | **2.25+0.12** | **1.88+0.08** | **2.16+0.13** | **1.35+0.07** | **1.32+0.09** | **1.57+0.06** |
| M2-1 | 0.70+0.01 | 1.68+0.02 | 2.58+0.11 | 1.02+0.06 | 0.70+0.04 | 1.73+0.11 | 0.41+0.04 | 2.89+0.01 | 2.86+0.04 | 0.71+0.00 | 1.28+0.04 |
| M2-2 | 0.28+0.09 | 1.43+0.08 | 1.83+0.02 | 0.71+0.06 | 0.49+0.06 | 1.36+0.07 | 0.54+0.05 | 2.46+0.04 | 1.66+0.05 | 0.84+0.07 | 0.92+0.03 |
| M2-3 | 0.41+0.05 | 1.15+0.03 | 2.24+0.04 | 1.16+0.08 | 0.63+0.09 | 2.32+0.20 | 0.47+0.05 | 3.89+0.19 | 2.53+0.26 | 0.77+0.04 | 0.87+0.26 |
| M2-4 | 0.17+0.01 | 0.79+0.02 | 1.20+0.00 | 0.48+0.01 | 0.49+0.10 | 1.14+0.05 | 0.36+0.04 | 2.84+0.32 | 1.10+0.01 | 0.55+0.03 | 0.47+0.02 |
| M2-5 | 0.39+0.03 | 2.04+0.07 | 2.01+0.22 | 1.36+0.17 | 57.73+3.45 | 3.17+0.07 | 0.41+0.01 | 3.25+0.29 | 1.84+0.25 | 0.79+0.05 | 0.86+0.04 |
| M2-6 | 0.98+0.23 | 3.19+0.15 | 2.11+0.21 | 0.92+0.14 | 38.33+2.77 | 3.33+0.29 | 0.55+0.00 | 5.33+0.46 | 1.78+0.18 | 0.99+0.17 | 0.54+0.06 |
| M2-7 | 0.14+0.05 | 0.93+0.21 | 0.85+0.02 | 0.37+0.00 | 0.77+0.08 | 1.13+0.00 | 0.54+0.05 | 1.84+0.06 | 0.75+0.05 | 0.48+0.04 | 0.76+0.01 |
| M2-8 | 0.23+0.00 | 2.43+0.29 | 1.27+0.14 | 0.47+0.03 | 0.78+0.14 | 1.58+0.11 | 0.40+0.01 | 4.18+0.09 | 0.74+0.04 | 0.49+0.00 | 0.95+0.07 |
| M2-9 | 0.46+0.05 | 2.60+0.22 | 3.86+0.10 | 1.44+0.02 | 0.95+0.10 | 2.82+0.08 | 0.65+0.05 | 4.92+0.02 | 3.49+0.15 | 1.16+0.00 | 1.58+0.09 |
| M2-10 | 0.31+0.15 | 1.29+0.09 | 1.60+0.05 | 0.64+0.01 | 0.76+0.08 | 1.55+0.07 | 0.45+0.03 | 3.73+0.30 | 1.62+0.04 | 0.68+0.11 | 1.08+0.15 |
| M2-11 | 0.30+0.02 | 0.75+0.03 | 1.90+0.05 | 0.74+0.01 | 0.54+0.02 | 1.74+0.20 | 0.47+0.05 | 2.29+0.00 | 1.33+0.09 | 0.44+0.07 | 1.14+0.06 |
| M2-12 | 0.18+0.01 | 0.49+0.07 | 1.17+0.02 | 0.36+0.02 | 0.54+0.01 | 1.60+0.07 | 0.47+0.04 | 1.80+0.06 | 0.93+0.02 | 0.46+0.03 | 0.67+0.04 |
| M2-13 | 0.27+0.01 | 0.91+0.02 | 1.59+0.07 | 0.79+0.01 | 1.37+0.01 | 1.15+0.12 | 0.71+0.03 | 2.47+0.03 | 1.43+0.07 | 0.72+0.02 | 1.18+0.02 |
| M2-14 | 0.41+0.04 | 1.44+0.02 | 1.76+0.09 | 0.58+0.00 | 0.93+0.05 | 1.53+0.02 | 0.65+0.05 | 3.71+0.08 | 1.93+0.27 | 0.93+0.09 | 0.99+0.09 |
| M2-15 | 0.54+0.04 | 1.32+0.01 | 2.04+0.07 | 1.14+0.02 | 0.34+0.02 | 2.66+0.08 | 0.27+0.02 | 7.22+0.20 | 1.83+0.05 | 0.64+0.03 | 1.03+0.09 |
| M2-16 | 0.34+0.01 | 0.97+0.18 | 1.36+0.13 | 0.41+0.12 | 0.77+0.02 | 2.33+0.01 | 0.41+0.04 | 4.67+0.01 | 1.00+0.02 | 0.54+0.06 | 0.65+0.01 |
| **MEAN(M2)** | **0.38+0.05** | **1.44+0.09** | **2.07+0.08** | **1.02+0.04** | **6.13+0.41** | **1.95+0.10** | **0.48+0.04** | **3.59+0.13** | **1.68+0.10** | **0.70+0.05** | **0.93+0.07** |
