# Supplementary material for: Laser Irradiation-Induced DNA Methylation Changes Are Heritable and Accompanied with Transpositional Activation of mPing in Rice
Source: Front Plant Sci. 2017 Mar 21;8:363. doi: 10.3389/fpls.2017.00363 (PMC5359294; doi:10.3389/fpls.2017.00363)
Supplement: Supplementary file 9 [file Table9.DOC]

Supplementary Table 9. Chromosomal location and predicted homology of the cloned DNA segments showing altertions in cytosine methylation based on MSAP

| Fragment | Sample | Chromosome  (BlastN) | Predicted homology  (BlastX) |
| --- | --- | --- | --- |
| MS-33 | g8-M6(26) | chr05; e-155 | NADH-glutamate synthase 2 precursor [Oryza sativa Japonica Group] |
| MS-62 | b3-H2(38) | chr02; 1e-082 | unspliced-genomic serine-threonine kinase receptor-associated protein, putative, expressed |
| MS-63 | b3-H8(32) | chr02; 6e-085 | unspliced-genomic serine-threonine kinase receptor-associated protein, putative, expressed |
| MS-64 | b4-H2(35) | chr07; 3e-041 | hypothetical protein OsI_26295 [Oryza sativa Indica Group] |
| MS-65 | b4-H4(35) | chr04; e-126 | transposon protein |
| MS-68 | b6-H11(39) | chr01; e-104 | hypothetical protein [Oryza sativa Japonica Group] |
| MS-71 | b8-H9(27) | chr04; e-128 | transposon protein |
| MS-72 | b10-H4(18) | chr04; 6e-094 | unspliced-genomic protein phosphatase 2C isoform gamma, putative, expressed |
| MS-73 | b10-H11(18) | chr01; e-104 | hypothetical protein [Oryza sativa Japonica Group] |
| MS-81 | b10-M11(18) | chr01; e-101 | hypothetical protein [Oryza sativa Japonica Group] |
| MS-83 | f4-H12(21) | chr04; 4e-043 | unspliced-genomic retrotransposon protein, putative, unclassified, expressed |
| MS-85 | f6-M12(28) | chr10; e-134 | CAF1 family ribonuclease containing protein [Oryza sativa (japonica cultivar-group)] |
| MS-87 | f8-H12(15) | chr02; 9e-030 | unspliced-genomic expressed protein |
| MS-89 | f8-M12(17) | chr01; e-161 | Os01g0136800 [Oryza sativa (japonica cultivar-group)] |
| MS-92 | g3-H12(31) | chr02  1e-076 | hypothetical protein OsI_07824 [Oryza sativa Indica Group] |
| MS-94 | g3-M12(28) | chr04; 5e-088 | hypothetical protein |
| MS-95 | g3-M12(31) | chr02; 2e-078 | unknown protein [Oryza sativa Japonica Group] |
| MS-96 | g4-H2(13) | chr06; 0.0 | unnamed protein product [Oryza sativa Japonica Group] |
| MS-99 | g4-H12(13) | chr08; 0.0 | hypothetical protein OsI_21375 [Oryza sativa Indica Group] |
| MS-100 | g4-H12(21) | chr07; e-147 | unspliced-genomic NHL repeat protein, putative, expressed |
| MS-103 | g4-M6(25) | chr01; 2e-094 | unspliced-genomic auxin-inducible protein, putative, expressed |
